# Molecular analysis of *FOXC1* in subjects presenting with severe developmental eye anomalies

**Published:** 2009-07-13

**Authors:** Kulvinder Kaur, Nicola K. Ragge, Jiannis Ragoussis

**Affiliations:** 1 Wellcome Trust Centre for Human Genetics, University of Oxford, Oxford, UK; 2 Department of Physiology, Anatomy and Genetics, University of Oxford, Oxford, UK; 3 Moorfields Eye Hospital, London and Birmingham Children’s Hospital, Birmingham, UK

## Abstract

**Purpose:**

Haploinsufficiency through mutation or deletion of the forkhead transcription factor, *FOXC1*, causes Axenfeld-Rieger anomaly, which manifests as a range of anterior segment eye defects and glaucoma. The aim of this study is to establish whether mutation of *FOXC1* contributes toward other developmental eye anomalies, namely anophthalmia, microphthalmia, and coloboma.

**Methods:**

The coding sequence and 3`-UTR of *FOXC1* was analyzed in 114 subjects with severe developmental eye anomalies by bidirectional direct sequencing.

**Results:**

Four coding *FOXC1* variations (two novel missense variations, one insertion, and one novel deletion) were identified in the cohort. Two noncoding variations were also identified in the 3′-UTR. The missense mutations were c.889C_T and c.1103C_A, resulting in p.Pro297Ser and p.Thr368Asn, respectively. The c.889C_T transition was identified in 19 of the 100 unaffected control samples. The c.1103C_A transversion resulted in a conservative substitution in an unconserved amino acid and was deemed unlikely to be pathogenic. A c.1142_1144insGCG change resulting in p.Gly380ins, which was previously associated with kidney anomalies, was identified in 44 of the 114 affected individuals. This variation was also present in 29 of the 87 unaffected controls and is therefore likely to be a polymorphism. A c.91_100delCGGCGGCCG deletion resulting in p.Ala31_33del was identified in one individual.  This deletion segregated with the moderately affected mother and unaffected maternal grandfather of the proband. This deletion was identified in one of the 307 unaffected controls.

**Conclusions:**

Our data suggests a potential susceptibility role for *FOXC1* in generating severe eye pathologies. However, on the basis of these results, it is unlikely that *FOXC1* mutation is a major causative factor of anophthalmia, microphthalmia, and coloboma.

## Introduction

Developmental eye anomalies (DEA) encompass a spectrum of severe structural defects of the eye caused by the disruption of the smooth process of ocular morphogenesis during early gestation [1]. With a birth prevalence of approximately 1 in 3,000–4,000, DEA are considered to account for at least 25% of childhood visual impairment worldwide [2,3]. The most severe forms of DEA are anophthalmia, characterized by the complete absence of ocular tissue in the orbit, and microphthalmia, which exhibits wide phenotypic variability and causes the eye to have an axial length of two standard deviations below the age-adjusted mean with variable intraocular abnormalities including coloboma [4].

A growing number of monogenic syndromes have begun to be identified in patients exhibiting DEA including those caused by mutations or deletions in orthodenticle homeobox 2 (*OTX2*), SRY (sex determining region Y)-box 2  (*SOX2*), H6 family homeobox 1 (*NKX5-3*), visual system homeobox 2 (*CHX10*), sonic hedgehog (*SHH*), retina and anterior neural fold homeobox (*RAX*), bone morphogenetic protein 4 (*BMP4*), BCL6 co-repressor (*BCOR*), chromodomain helicase DNA binding protein 7 (*CHD7*), and paired box 6 (*PAX6*) [5-16]. Furthermore, there are up to 400 different chromosome aberrations described in the various dysmorphology databases including deletions, duplications, and translocations [17-23]. Many of these aberrations are likely to act by disrupting eye development genes and likely to harbor additional candidate genes that can be studied to further understand developmental eye disorders. One particular example is the 6p25 deletion syndrome. This syndrome causes deafness [24], developmental delay, facial dysmorphology [25], brachycephaly, schizophrenia [26], and anterior eye anomalies [23-27]. The eye anomalies are thought to be due to a deletion of the forkhead box C1 gene (*FOXC1*).

FOXC1 is a member of the forkhead family of transcription factors, characterized by their molecular arrangement of two wings connecting β strands flanking one of three α helices [28]. This helix-turn-helix structure comprises the evolutionarily conserved forkhead domain of 110 amino acids through which the FOX proteins are able to interact with DNA and translocate to the cell nuclei [29]. Forkhead genes act as critical regulators of embryogenesis, cell migration, and cell differentiation. Disruptions within the *FOX* genes have long been associated with pathogenicity and ocular disease in particular [28]. *FOXC1* whole gene deletions or mutations within or affecting the forkhead domain through which FOX proteins are able to interact with DNA and translocate to the cell nuclei [29] underlie Axenfeld-Rieger anomalies. To date, at least 30 different missense, nonsense, and frameshift mutations have been identified, affecting the forkhead domain of FOXC1 in individuals presenting with the spectrum of ocular defects associated with Axenfeld-Rieger syndrome and anomaly (anteriorly-displaced Schwalbe’s line, iris adhesions, iridocorneal angle dysgenesis, and corectopia [30-44]). Approximately half of these patients also develop glaucoma, which may cause further visual deterioration. Interestingly, both duplications and deletions of the 6p25 segment containing *FOXC1* are associated with anterior eye malformations [25,45]. These seemingly complex genotype-phenotype associations are consistent with *FOXC1* gene dosage effects [46]. Intriguingly, one such study by Gould et al. [23] describes seven individuals with 6p25 deletion syndrome associated with ocular dysgenesis of which two individuals presented with microphthalmia.

Since deletions of *FOXC1* have been associated with microphthalmia [23], an investigation into the role of *FOXC1* in producing developmental eye anomalies, distinct from those associated with Axenfeld-Rieger syndrome, is important in enabling us to delimit the effect of this gene. We therefore decided to investigate a wider role for *FOXC1* in underlying developmental eye anomalies and screened the gene for disease-causing variations in a cohort of patients exhibiting anophthalmia, microphthalmia, and coloboma.

## Methods

One hundred and fourteen subjects with developmental eye anomalies consisting of unilateral microphthalmia with contralateral normal eye or minor defect such as myopia (n=33); bilateral microphthalmia (n=20) including one with bilateral Peter’s anomaly and one with anterior segment dysgenesis; bilateral anophthalmia (n=12); unilateral anophthalmia with contralateral defect e.g., retinal dystrophy (n=7); unilateral anophthalmia with contralateral coloboma (n=2); unilateral anophthalmia and contralateral microphthalmia (n=3); unilateral anophthalmia with contralateral normal eye or minor defect e.g., myopia (n=11); unilateral coloboma with contralateral normal eye (n=3); unilateral microphthalmia with bilateral coloboma (n=1); unilateral microphthalmia with unilateral coloboma (same eye; n=8); unilateral microphthalmia with contralateral coloboma (n=4); bilateral coloboma (n=2); bilateral microcornea (n=1); unilateral microphthalmia with contralateral defect e.g., retinal dystrophy (n=5); bilateral Peter’s anomaly with normal sized eyes (n=1); and unilateral Peter’s anomaly (n=1) were screened for variations in the coding region of *FOXC1* (Ensembl Transcript: FOXC1–001 ENST00000380874). These individuals had been previously screened for mutations in genes known to be associated with anophthalmia, microphthalmia, and coloboma, including *SOX2, OTX2, SHH*, and *BMP4* [5,13]. Informed consent was obtained from all subjects under full ethics approval as previously described [5,13]. Familial DNA and ethnically matched control DNA samples were obtained where possible in the event of a putative causative variant being identified. A control cohort of 100 Yoruba people, 87 CEPH (Centre d'Etude du Polymorphisme Humain) samples, and 307 British Caucasians were analyzed for the variations p. Pro297Ser, p.Gly380ins, and p. Ala31_33del, respectively. Statistical analysis comprising a Fisher's exact test for count data was performed on the data sets using the R software package.

Eight primer pairs spanning the exonic sequence and 3`-UTR of *FOXC1* were designed using Primer3 (Table 1) and amplified by polymerase chain reaction (PCR) on a DNA Thermocycler 9700 (Applied Biosystems®, Foster City, CA). PCR was performed according to the manufacturer’s standard protocol in 10 µl reaction volumes using the FailSafe^TM^ PCR System (EPICENTRE® Biotechnologies, Madison, WI) for amplicons FOXC1i, FOXC1ii, and FOXC1iii and Qiagen HotStarTaq DNA Polymerase (Qiagen®, Valencia, CA) for amplicons FOXC1iv, FOXC1v, FOXC1vi, FOXC1vii, and FOXC1viii under the reaction conditions detailed in Table 1.

**Table 1:** *FOXC1* PCR amplifications and primer details.

**Amplicon**	**Reaction conditions**	**Annealing temperature**	**Forward primer sequence**	**Reverse primer sequence**	**Fragment length (bp)**	**Genomic position**	**Region**
FOXC1i	FailSafe – G	56.5	CGGTTCTCACCTCCCATTG	TTGACGAAGCACTCGTTGAG	1045	chr6: 1555048–1556092	Exonic
FOXC1ii	FailSafe – K	60	AGTTCATCATGGACCGCTTC	ACGTACCGTTCTCGGTCTTG	381	chr6: 1555996–1556376	Exonic
FOXC1iii	FailSafe – J	61	GCATCCAGGACATCAAGACC	CAAGTGGCCCAGGTCTCC	791	chr6: 1556344–1557134	Exonic
FOXC1iv	Qiagen	60	CTCACCTCGTGGTACCTGAAC	AGAGTTTTCTTCGTGCTGGTG	441	chr6: 1557087–1557527	Exonic/ 3′-UTR
FOXC1v	Qiagen	60	TCCCTCCAAAAATTCAGCTC	ACGTCAGGTTTTGGGAACAC	434	chr6: 1557482–1557915	3′-UTR
FOXC1vi	Qiagen	60	TGGATGTCGTGGACCAAAC	CTAGCCTCAAAGCAAGCTGAC	414	chr6: 1557869–1558282	3′-UTR
FOXC1vii	Qiagen	59	TTTATTTTCCTGCAGCATCTTC	GATTAAATATCCCTTTCCAACC	462	chr6: 1558222–1558680	3′UTR
FOXC1viii	Qiagen	59	TCCCCCATTTACAATCCTTC	AATCACAGGCCACGTAGAGC	557	chr6: 1558597–1559153	3′UTR

Reaction conditions indicate the PCR mix used according to the manufactures standard protocols of the specified kits. The genomic position quoted is the position within Ensembl Transcript: FOXC1-001 ENST00000380874.

Sequencing reactions were performed with the PCR primers on ExoSAP-IT® cleaned products (USB Corporation, Cleveland, OH) using BigDye^TM^ Terminator (v.3.1) Cycle Sequencing Ready Reaction Kit (Perkin-Elmer, Waltham, MA) and resolved on an ABI Prism Genome Analyzer 3700 or 3100 for longer reads (Applied Biosystems®). Data were analyzed by constructing contigs aligned to a reference sequence using Sequencher^TM^ software (v.4.5; Gene Codes Corporation, Ann Arbor, MI). Confirmation of the sequence changes was obtained using a second sample.

## Results

**Figure 1:**
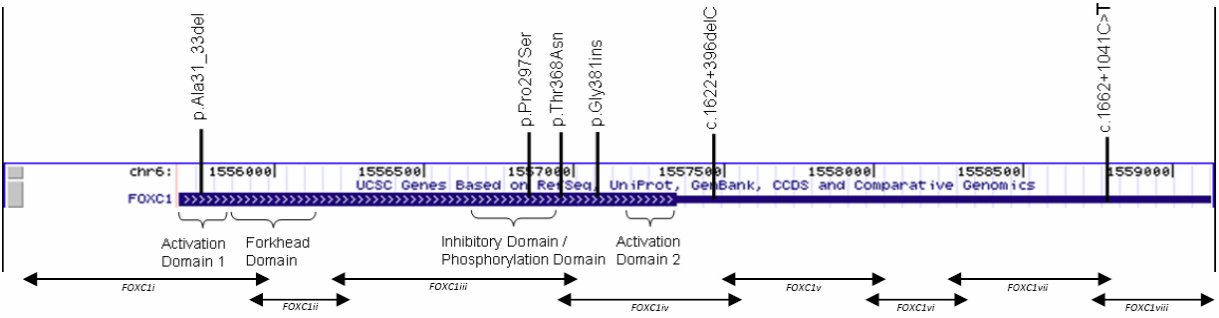
FOXC1 gene structure. Locations of amplicons and identified variations are displayed.

Four *FOXC1* coding sequence alterations were revealed by direct sequencing comprising two missense variations, one insertion, and one deletion. Two noncoding variations were also identified (Table 2 and Figure 1).

**Table 2:** Detected variations and disease phenotype.

**Variation**	**Phenotype**
Heterozygous p.Pro297Ser	Unilateral microphthalmia and sclerocornea
Heterozygous p.Pro297Ser	Unilateral extreme microphthalmia with cyst, contralateral myopia
Heterozygous p.Thr368Asn	Unilateral microphthalmia and dense cataract
Heterozygous p.Gly380ins	Multiple patients
Homozygous p.Gly380ins	Multiple patients
Heterozygous p.Ala31_33del	Right optic disc coloboma and left iris and chorioretinal coloboma
Heterozygous 3`-UTR 1662+1041C>T	Right optic disc coloboma and left iris and chorioretinal coloboma
Heterozygous 3`-UTR 1622+396delC	Bilateral microphthalmia with Rieger anomaly and
Heterozygous 3`-UTR 1622+396delC	Unilateral microphthalmia with microcornea and subtotal retinal detachment

All variations detected within the patient cohort with their corresponding phenotype.

**Figure 2:**
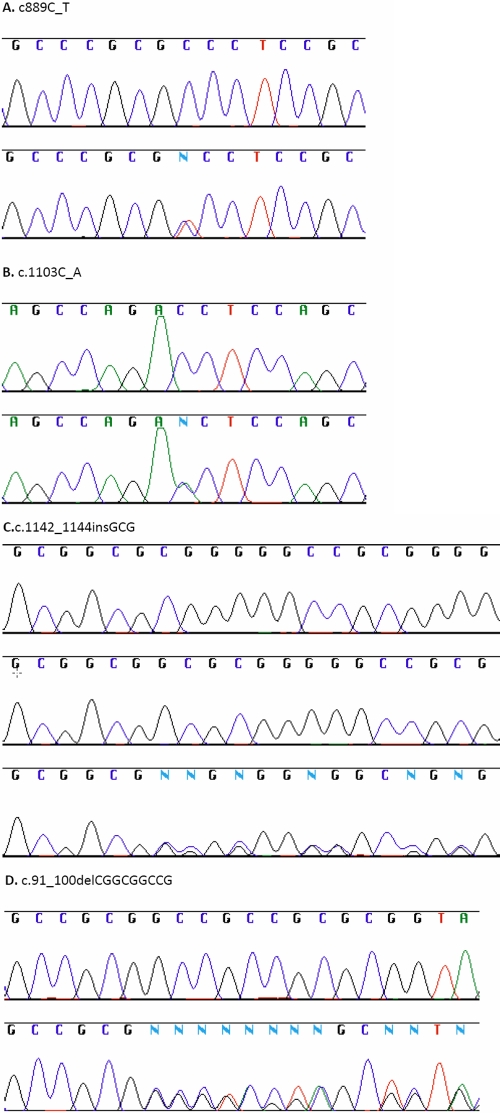
Direct sequencing analysis of the coding region of *FOXC1*. **A**: The lower sequence shows the heterozygous missense mutation c889C_T (p.Pro297Ser) identified in two individuals, and the upper sequence is the corresponding wild-type sequence. **B**: The lower sequence shows the heterozygous missense mutation c.1103C_A (p.Thr368Asn) detected in one individual, and the upper sequence is the corresponding wild-type sequence. **C**: The bottom sequence shows the 3-bp heterozygous insertion mutation, c.1142_1144insGCG (p.Gly380ins), detected in 37 individuals. The middle sequence shows the equivalent homozygous insertion detected in seven individuals. The upper sequence displays the corresponding wild-type sequence. **D**: The lower sequence shows the 9-bp heterozygous deletion, c.91_100delCGGCGGCCG (p.Ala31_33del), detected in the proband, mother, maternal grandfather, and 1 of the 307 control samples, and the upper sequence displays  the corresponding wild-type sequence.

The deletion (c.91_100delCGGCGGCCG; Figure 2D) caused a three residue contraction of the alanine tract (p.Ala31_33del), located 41 amino acids upstream of the forkhead domain in activation domain 1 of the protein [29]. The deletion resulted in the removal of a SacII recognition site from the PCR product. This deletion was identified in one individual who presented with an optic disc coloboma in both eyes with additional iris and chorioretinal coloboma in the left eye. The moderately affected mother and unaffected maternal grandfather were all heterozygous for the deletion. Three hundred and seven ethnically matched control DNA samples of British Caucasian origin and the family members of the proband (parents, maternal grandparents, sister) were screened for the variation using a restriction digest method exploiting the absence of the SacII restriction site. One control sample was also heterozygous for c.91_100delCGGCGGCCG. These frequencies were analyzed using Fisher’s exact test and found not to be significant (p=0.4678)

The first missense mutation was present in two patients, one of Nigerian and the other of Hispanic descent. It resulted from a c.889C_T transition (Figure 2A), causing a p.Pro297Ser amino acid alteration in the protein inhibitory/phosphorylation domain (as defined by Berry et al. [29]). This variation resulted in the introduction of an HgaI restriction site into the gene. One hundred ethnically matched DNA samples of Yoruba descent were screened for the variation in a restriction digest assay with HgaI. The variation was identified in 19 of the control samples.

The second missense mutation, c.1103C_A (Figure 2B), resulted in p.Thr368Asn outside any of the defined functional domains of the protein. This variation was present in a patient of Singaporean-Filipino descent and resulted in a conservative amino acid substitution in a residue, which was highly variable across species. No familial or ethnically matched control DNA sample was available for this variant and thus could not be investigated further. SIFT (Sorting Intolerant From Tolerant) analysis [47] supported the low likelihood of p.Thr368Asn being a pathogenic variant.

The final variation identified was a c.1142_1144insGCG change (Figure 2C), resulting in p.Gly381ins, which was present in a heterozygous form in 37 individuals and a homozygous form in seven individuals. This variation has been previously reported in a screen of patients with congenital abnormalities of the kidney and urinary tract [48]. The heterozygous form of this variation was identified in 27 out of 87 unaffected control samples and was present as a homozygous variant in two of the control samples. Fisher’s exact test analysis of these frequencies indicated that they were not significant (p=0.4523)

## Discussion

We report the first screen of patients manifesting with severe developmental eye anomalies for disease-causing variations in *FOXC1*. Since deletions of *FOXC1* have been associated with microphthalmia [23], an investigation into the role of *FOXC1* in producing developmental eye defects distinct from those previously associated with the mutation in this gene is important to enable us to delimit its effect. To date, mutations of human *FOXC1* have been associated with anterior segment dysgenesis, iris anomalies, and developmental glaucoma. These phenotypes may arise from an abnormality in the migration and/or differentiation of mesenchymal cells that contribute to the anterior segment of the eye as in the mouse. Kume et al. [49] demonstrated that 16.5 dpc (days post coitum) *Foxc1* null mice display several ocular anomalies including a disorganized arrangement of cells in the cornea, iris hypoplasia, unfused eyelids, and a reduced number of mesenchymal cells in the future stromal region.

Although an important role for *FOXC1* in eye development is clear, the complexities of this association are evident through the lack of any conclusive genotype-phenotype correlations; the eye phenotypes associated with 6p25 deletion syndrome often exhibit variable penetrance [25]. Individuals with *FOXC1* mutations or deletions also demonstrate a spectrum of phenotypic consequences, including the mutated allele segregating with affected and unaffected members of the same family [36,39-41]. It has therefore been suggested that either environmental factors and/or modifier genes interact with *FOXC1* in producing a disease phenotype [50], and this is not uncommonly seen with other ocular developmental genes [13].

The level of phenotypic variability could also be attributed to stochastic factors in development related to spatio-temporal events and the level of expression of developmentally important downstream targets of *FOXC1* [50]. Recent studies have identified one of those downstream targets as another forkhead transcription factor, *FOXO1A* [51]. The zebrafish *foxO1a* ortholog is strongly expressed in the periocular mesenchyme, and its expression pattern is significantly reduced in a *foxc1* siRNA knocked down model. The reduced *foxc1* expression increases cell death in the developing zebrafish eye and demonstrates a novel role for *FOXC1* as an essential mediator of cellular homeostasis in the eye.

Unaffected control DNA analysis of c.889C_T demonstrated a high likelihood of this variation being a common polymorphism in the Yoruba population with the variant segregating with 19 out of 100 control samples. The second missense mutation, c.1103C_A, was not analyzed further due to the low likelihood of it being a pathogenic variant.

The c.1142_1144insGCG insertion had been previously reported in a study of patients with congenital abnormalities of the kidney and urinary tract [48]. This variation was present in three of the seven patients presented in the study and was postulated to be causative for the disease phenotype. The identification of this variation in 29 of the 87 controls screened in our study suggests that this variation is a non-pathogenic polymorphism.

The most interesting variation identified in our screen was the deletion resulting in the contraction of the alanine tract to three residues by the deletion. The alanine tract is originally already very short, consisting of only six residues. A polyalanine tract upstream of the forkhead domain is a feature common to all of the forkhead transcription factors and variations in the length of polyalanine repeats have previously been demonstrated to underlie disease phenotypes, e.g., *FOXL2* in blepharophimosis-ptosis-epicanthus inversus syndrome (BPES) [52] and *FOXE1* in thyroid dysgenesis [53]. In the *FOXE1*/thyroid dysgenesis model, there is a significant association between a shorter polyalanine tract and the manifestation of the disease phenotype. As a disease-causing mechanism, Carré et al. [53] demonstrate that the transcriptional activation of *FOXE1* with 16 alanines is significantly higher than *FOXE1* containing 14 alanines. They concluded that *FOXE1* significantly modulates the risk of thyroid dysgenesis occurrence through its alanine-containing stretch and proposed a mechanism linking the polyalanine tract containing transcription factors to disease. Interestingly, the disease-associated variant in *FOXE1* also segregates with unaffected controls, which is similar to our observation with the contraction of the polyalanine tract in *FOXC1*. Therefore, this is consistent with the contraction of alanine tract being a susceptibility factor rather than a disease-causing mutation.

The oligogenic basis of developmental eye anomalies is well recognized and will only be resolved when a comprehensive candidate gene set has been analyzed for mutations, coding polymorphisms, or copy number variations. In this study, we have identified several variations affecting the coding sequence of *FOXC1*, some of which could contribute to phenotype severity and penetrance. Although a direct causative role for *FOXC1* mutations in our cohort of patients with developmental eye anomalies has not been definitively shown, *FOXC1* could contribute genetic susceptibility either through contraction in the length of the polyalanine tract or other genetic variations similar to the ones described here.
